# *In silico* profiling of analgesic and antihyperglycemic effects of ethanolic leaves extract of *Amischotolype mollissima*: Evidence from *in vivo* studies

**DOI:** 10.1016/j.sjbs.2022.103312

**Published:** 2022-05-20

**Authors:** Maisha Maliha Medha, Hiron Saraj Devnath, Biswajit Biswas, Bishwajit Bokshi, Samir Kumar Sadhu

**Affiliations:** a Pharmacy Discipline, Life Science School, Khulna University, Khulna 9208, Bangladesh; b Department of Pharmacy, Jashore University of Science & Technology, Jashore 7408, Bangladesh

**Keywords:** *Amischotolype mollissima*, Commelinacea, Antioxidative, Analgesic, Antihyperglycemic, GC-MS analysis, *In silico* profiling, ANOVA, one-way analysis of variance, bw, body weight, GC–MS, gas chromatography-mass spectroscopy, SC_50_, scavenging concentration 50%, NIST, National Institute of Standards and Technology, PDB ID, protein data bank identification code, SD, standard deviation, UV, ultra-violet

## Abstract

The aim of this study is to assess the antioxidative profile and related pharmacological potentialities of the ethanolic extract of *Amischotolype mollissima* leaves, traditionally used in treating pain, injury, malarial fever, epilepsy and hyperacidity, followed by a computational approach for the analysis of bioactive compounds identified by GC–MS. In GC–MS analysis, the extract yielded ten compounds, with 4,6-di-t-butyl-2-alpha-methyl benzyl phenol having the highest amount. *In vitro* investigation of the antioxidative properties of the plant was conducted with 2,2-diphenyl-1-picryl hydrazyl (DPPH) radical and hydrogen peroxide scavenging assays. The amounts of secondary metabolites phenolics, flavonoids, and tannins were measured at 142 mg GAE/g, 534 mg QE/g, and 110 mg GAE/g, respectively. An acute toxicity study was carried out on mice, which revealed no toxicity up to the dosage of 4000 mg/kg bw. For the dosages of extract at 250 and 500 mg/kg bw, the writhing response test induced by acetic acid exhibited a statistically significant (p < 0.05) analgesic effect in mice. The oral glucose tolerance test (OGTT) and alpha-glucosidase enzyme inhibitory activity assay were used to examine the antihyperglycemic potential, in which the extract reduced the blood glucose level to 6.22 mmol/l and 3.82 mmol/l, at dosages of 250 and 500 mg/kg bw, respectively at 60 min in OGTT even though no activity was observed in the α-glucosidase enzyme inhibitory assay. In an antibacterial assay, the extract's minimum inhibitory concentration (MIC) against *E. coli*, *P. aeruginosa*, and *S. aureus* was determined to be 8, 16, and 8 µg/ml, respectively. This study shows that the usage of *A. mollissima* leaves in folklore medication is justified.

## 1 Introduction

Traditional medicine has relied on natural products for thousands of years (Newman, 2008). Original natural products, products generated semisynthetically from natural sources, and synthetic products are the three types of natural products. These plant-based natural products obtained from plants and used by indigenous people have been utilized to derive quite several important modern drugs, even with the mobilization of modern and allopathic medicines (Balick et al., 1996). Drugs such as aspirin, atropine, ephedrine, digoxin etc. are examples of pharmaceuticals used in modern medicine, which were discovered by investigating their traditional use (Gilani, 2005). Hence, research into natural sources of drugs can be beneficial for identifying novel compounds with significant pharmacological effects and minimal side effects.

A number of biochemical reactions in our physiological system give rise to free radicals like reactive oxygen species (ROS) and reactive nitrogen species (RNS) (Mongalo et al., 2018). These reactive species are the by-products of aerobic metabolism, which involve the mitochondrial electron transport chain, nitric oxide synthases, NADPH oxidases, nitrite reductases etc. (Smallwood et al., 2018). Oxidative stress due to excessive ROS production can cause depletion of intracellular antioxidants, resulting in lipid peroxidation (Mao et al., 2020) and eventually leading to multiple diseases such as diabetes, atherosclerosis, inflammatory conditions, cancer etc. (Oğul et al., 2021). As there may be a variation in different antioxidant testing systems (Fawzi Mahomoodally et al., 2018), the antioxidant potential of *A. mollissima* extract was determined by both DPPH radical and hydrogen peroxide scavenging assays.

Diabetes mellitus is a widespread chronic metabolic disorder attributed to hyperglycemia due to hereditary or acquired insulin deficiency or resistance, along with changes in carbohydrate, fat and protein metabolism. It causes an impairment of glucose homeostasis, thereby resulting in various pathological conditions such as nephropathy, retinopathy, neuropathy, and neurological disorders (Kifle et al., 2020a). Although the current treatment strategies for diabetes mellitus include combination or monotherapy with antidiabetic agents of the classes thiazolidinediones, sulfonylureas, α-glucosidase and α-amylase inhibitors, these therapies are not devoid of harmful side effects (Kifle and Belayneh, 2020). According to World Health Organization (WHO), there are 21,000 medicinal plants extensively used for their pharmacological effects in the world, among which a growing number of bioactive compounds derived from plant sources have been identified to possess certain potential in the treatment of diabetes (Rizvi and Mishra, 2013).

Algesia or pain is referred to as an experience involved with actual or potential tissue injury, which is unpleasant to perceive in sensory and emotional manners (Raja et al., 2020). It plays an important adaptive function by alerting us in case of impending harm upon detection of noxious stimuli, and recovery from injury is facilitated by the elevated sensitivity related to inflammatory pain (Prescott and Ratté, 2017). Prostaglandins, proinflammatory cytokines, and chemokines are inflammatory mediators that cause pain by directly activating nociceptors, the primary sensory neurons that detect noxious stimuli (Matsuda et al., 2019). Presently, it is essential to develop novel strategies for pain relief in order to obtain antinociceptive agents with greater analgesia at lowered dosages and fewer side adverse effects (Naghizadeh et al., 2016). As a result, there is a need to look for alternative therapies sourced from plant origin that can alleviate inflammation and pain.

The perennial erect herb *Amischotolype mollissima* (family- Commelinaceae) is a flowering plant with fibrous roots, oblanceolate leaves and pink flowers. This species is distributed in India, Bangladesh, Singapore, Malaysia and Indonesia. It occurs in the Chittagong and Sylhet hill tracts of Bangladesh. It is found in wet and evergreen broad-leaved forests (Nandikar and Gurav, 2014). Traditionally, the paste made from *A. mollissima* leaves was found beneficial in treating malarial fever, epilepsy, hyperacidity and traumatic injury (Rudra et al., 2021). Further evidence from indigenous people showed that the whole plant is used in food poisoning, obstetric diseases or body pain, epilepsy, hyperacidity and traumatic injury (Srithi et al., 2009) and as an antiseptic (Dubost et al., 2019). In this study, the phytochemistry and pharmacological activities of *A. mollissima* were investigated through *in vitro, in vivo* and *in silico* studies to determine scientific evidence of its ethnopharmacological uses.

## 2 Materials and methods

### 2.1 Collection and preparation of plant

Leaves of *A. mollissima* were acquired from hill tracts of Sylhet in September 2018 and verified by the specialists of National Herbarium, Dhaka, Bangladesh. A specimen of the plant was stored in the institute as a voucher specimen for future reference (reference no. DACB: 46791). The leaves were separated, dried in shade, and ground into a coarse powder. 200 gm dried plant powder was macerated by 1.3 liter ethanol for 7 days at room temperature. Upon filtration, the marc was macerated for further 7 days using the same volume of solvent for exhaustive extraction of plant material. A total extract of 7.2 gm was obtained from 200 gm of dried powder by concentrating the filtrate using a rotary evaporator, making the yield of crude extract for *A. mollissima* to be 3.6 % w/w.

### 2.2 Chemicals

Analytical grade chemicals such as acetic acid, chloroform, ethanol, methanol, sulfuric acid, powdered glucose, mercuric iodide, nitric acid, potassium sodium tartrate, anhydrous sodium carbonate, sodium hydroxide, iodine, potassium dichromate, bismuth nitrate and ammonia solution were all obtained from Merck, Germany. Tartaric acid, picric acid, lead acetate, potassium dihydrogen phosphate and ferric chloride were supplied by Loba, India. Other reagents include distilled water, castor oil, potassium iodide (Sigma-Aldrich, Germany), DPPH (Sigma-Aldrich, USA), alpha-naphthol (Unichem, China), cupric sulfate (Qualikems, India), sodium bicarbonate (Guangdong Guanghua chemical factory, China), polysorbate-80 (Research-Lab Fine Chem Industries, India), alpha-glucosidase enzyme from *Saccharomyces cerevisiae*, *para*-nitrophenyl-α*-*D-glucopyranoside (*p*NPG) (Sigma-Aldrich, Switzerland). Standard drugs diclofenac sodium, glibenclamide and voglibose used in this study were procured from Beximco Pharmaceuticals Ltd., Bangladesh.

### 2.3 Animals

Swiss-albino mice (Mus musculus) with an average weight of 16–22 gm were purchased from the Animal House, Jahangirnagar University, Dhaka, Bangladesh. Khulna University Research Cell approved the animal ethics application (ref. no. KUAEC-2020/09/15) and all the rules and guidelines were followed while conducting *in vivo* experiments.

### 2.4 Phytochemical content

#### 2.4.1 Preliminary phytochemical screening

For complete phytochemical profiling of the plant, preliminary qualitative phytochemical tests were conducted on the *A. mollissima* leaves extract for evaluation of the chemical nature of the extract (Khalid et al., 2018).

#### 2.4.2 GC–MS analysis

The phytochemicals in the extract were determined using GC–MS according to a previously published procedure, in comparison to the NIST database (Rahman et al., 2021). The relative quantity of each component was reported as a percentage of peak area upon comparison with the total peak area.

#### 2.4.3 Estimation of total phenolic content (TPC)

TPC was studied following the Folin-Ciocalteu (FC) technique, where different concentrations (0.15–0.02 mg/ml) of standard gallic acid were used (Debnath et al., 2020). Using the gallic acid calibration curve, TPC was calculated as mg of gallic acid equivalent (GAE)/gram of dry extract.

#### 2.4.4 Estimation of total flavonoid content (TFC)

TFC was measured by the aluminum chloride colorimetric method (Biswas et al., 2018). TFC was denoted as mg of quercetin equivalent (QE)/ gram dry extract following the quercetin calibration curve.

#### 2.4.5 Estimation of total tannin content (TTC)

TTC was measured with the aid of the FC method (Debnath et al., 2020). The absorbance of the sample, standard and blank solutions was recorded at 725 nm. TTC was denoted as mg GAE)/gram of dry extract with help of the gallic acid calibration curve.

### 2.5 Antioxidative properties

#### 2.5.1 Thin layer chromatography (TLC) based qualitative antioxidant assay

The extract was subjected to a qualitative antioxidant test using the thin layer chromatographic technique (Sadhu et al., 2003).

#### 2.5.2 DPPH radical scavenging assay (DRSA)

*A. mollissima* extract was assessed for the free radical scavenging capacity in accordance with the procedure of (Biswas et al., 2018) with slight modification. DPPH was weighed and dissolved in methanol to make a 0.008% w/v solution. Various concentrations of the standard ascorbic acid and *A. mollissima* extract in methanol solution (100 μl) were taken in wells followed by the addition of 100 µl DPPH dissolved in methanol to each well. The percentage of DPPH radical scavenging of both *A. mollissima* extract and ascorbic acid was determined following the formula:

$$\text{DPPH}\;\;\text{radical}\;\;\text{scavenged}\;\left(\%\right)=[\left({\text{OD}}_{\text{C}}-{\;\text{OD}}_{\text{S}}\right)/{\text{OD}}_{\text{C}}]\times \;\text{1}00$$

where OD_C_ is the absorbance of the control and OD_S_ is the absorbance of the sample or standard. SC_50_ was determined from the % inhibition vs. log concentration graph.

#### 2.5.3 Hydrogen peroxide scavenging assay (HPSA)

The hydrogen peroxide scavenging capacity of *A. mollissima* extract was measured by colorimetric assay (Golder et al., 2020). The percentage of hydrogen peroxide scavenging of *A. mollissima* extract and ascorbic acid was determined following the formula:

$${\text{H}}_{\text{2}}{\text{O}}_{\text{2}}\;\text{scavenged}\;\;\left(\%\right)=\left[\left({\text{A}}_{\text{o}}-{\;\text{A}}_{\text{1}}\right)/{\text{A}}_{\text{o}}\right]\times \text{1}00$$

where A_o_ is the absorbance of the control and A_1_ is the absorbance of the sample or standard. SC_50_ was determined from the % inhibition vs. log concentration graph.

#### 2.5.4 Determination of total antioxidant capacity (TAC)

The total antioxidant capacity of *A. mollissima* extract was evaluated by the phosphomolybdate method, with ascorbic acid as the standard (Umamaheswari and Chatterjee, 2008). 0.3 ml of extract and ascorbic acid and blank (ethanol) were combined with 3 ml of reagent mixture (0.6 M sulphuric acid, 28 mM sodium phosphate and 4 mM ammonium molybdate) separately and incubation was carried out at 95 °C for 90 min. Following the cooling of the samples, the absorbance of the mixture was taken at 695 nm. The TAC of the extract in ascorbic acid equivalents (AAE) was measured following the equation:

TAC(mg AAE/g)=AAE/sampleconcentration(gm/ml)

### 2.6 Acute toxicity study

The acute toxicity study was conducted in mice following the guidelines 423 of the Organization for Economic Co-operation and Development (OECD, 2002). Four groups of mice were treated with the 400, 1000, 2000 and 4000 mg/kg bw of the extract, and their general behavior, adverse effects, mortality, and changes in body weight were determined in comparison to the normal group. Observations were conducted individually after dosing during the first 30 min, periodically in the first 24 h, and daily for the next 14 days.

### 2.7 Analgesic activity study

To evaluate the analgesic activity, the acetic acid-induced abdominal writhing model was used (Saha et al., 2021). The percentage inhibition of writhing behavior was determined with comparison to the control group following the formula:

$$\text{Inhibition}\;\;\text{of}\;\;\text{writhing}\;\;\left(\%\right)=\left[\left({\text{W}}_{\text{C}}-{\;\text{W}}_{\text{T}}\right)\times \;\text{1}00\right]/{\;\text{W}}_{\text{C}}$$

where W_C_ = mean writhing number of the control group and W_T_ = mean writhing number of test groups.

### 2.8 Antimicrobial activity assay

The antimicrobial activity of the extract was assessed against *Escherichia coli*, *Pseudomonus aeruginosa* and *Staphylococcus aureu*s bacterial strains by determining their respective MICs using the broth dilution technique (Balouiri et al., 2016). The lowest concentration of the extract which showed no turbidity indicating no visible growth when compared to control tubes was regarded to be the minimum inhibitory concentration (MIC).

### 2.9 Evaluation of antihyperglycemic activity

#### 2.9.1 Oral glucose tolerance test (OGTT)

The antihyperglycemic potential of the extract was examined through OGTT in mice (Debnath et al., 2021) and their fasting blood glucose level was noted at 0, 30, 60 and 90 min after the administration of glucose solution (2 gm/kg bw). Then, the capacity of the extract to lower blood levels of glucose was compared to that of the standard glibenclamide and the control group.

#### 2.9.2 Alpha-glucosidase enzyme inhibitory activity assay

The inhibitory effect of the extract on the alpha-glucosidase enzyme was determined following the standard method after some minor modifications (Telagari and Hullatti, 2015). Various concentrations (0.1–0.5 mg/ml) of voglibose were taken as standard The result was denoted as percentage inhibition following the formula:

$$\text{Inhibitory}\;\;\text{activity}\;\left(\%\right)=\left(\text{1}\;-{\;\text{A}}_{\text{S}}/{\text{A}}_{\text{C}}\right)\times \;\text{1}00$$

where A_S_ is the absorbance in the test solution and A_C_ is the absorbance of control.

### 2.10 *In silico* analysis*:*

#### 2.10.1 Ligand preparation:

Three-dimensional structures of the major compounds identified in GC–MS analysis were obtained from PubChem (https://pubchem.ncbi.nlm.nih.gov/) to be used as ligands. Few 2D structures of the ligands were drawn in the ChemDraw 3D 15.0 (Cambridge soft corporation). After that, energy minimization was done by PyRx (Hanwell et al., 2012).

#### 2.10.2 Protein preparation:

The protein data bank (https://www.rcsb.org) was used to determine the protein structures (PDB ID: 4NOS and PDB ID: 6JB3). Discovery studio visualizer was downloaded to clean the proteins and add polar hydrogen (version 20.1.0.19295) (Systèmes, 2020). Energy optimization was conducted using SwissPDB viewer (Guex and Peitsch, 1997).

#### 2.10.3 Molecular docking and visualization:

Active site amino acid residues were selected while docking was performed *via* autodock vina 4.2 in PyRx (Dallakyan and Olson, 2015). The result was analyzed using ligplot plus version 2.2.4 (Wallace et al., 1995).

### 2.11 Statistical analysis

Mean ± SD was used to express the data. The results were interpreted using SPSS 16 through ANOVA, where p < 0.05 was considered statistically significant. The graphs were drawn using GraphPad Prism 8.0.2 (GraphPad Software Inc., San Diego, CA).

## 3 Results

### 3.1 Phytochemical profile

The phytochemical tests in the preliminary phytochemical screening showed that phenols, tannins, flavonoids, glycosides, terpenoids and carbohydrates were found in the ethanolic *A. mollissima* extract*.*

**Fig. 1:**
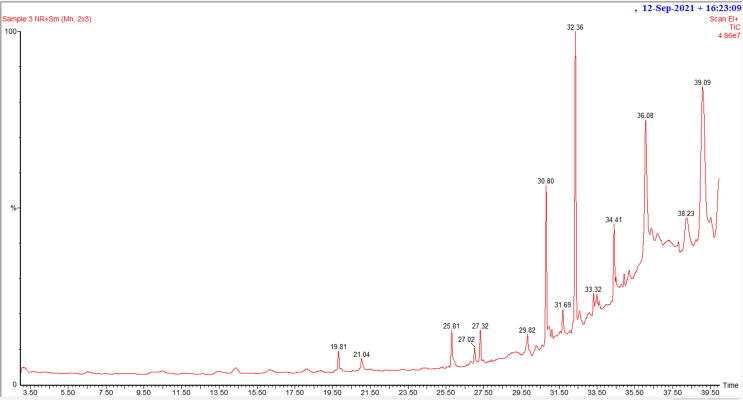
GC–MS-based chromatogram of *A. mollissima* leaves extract.

**Fig. 2:**
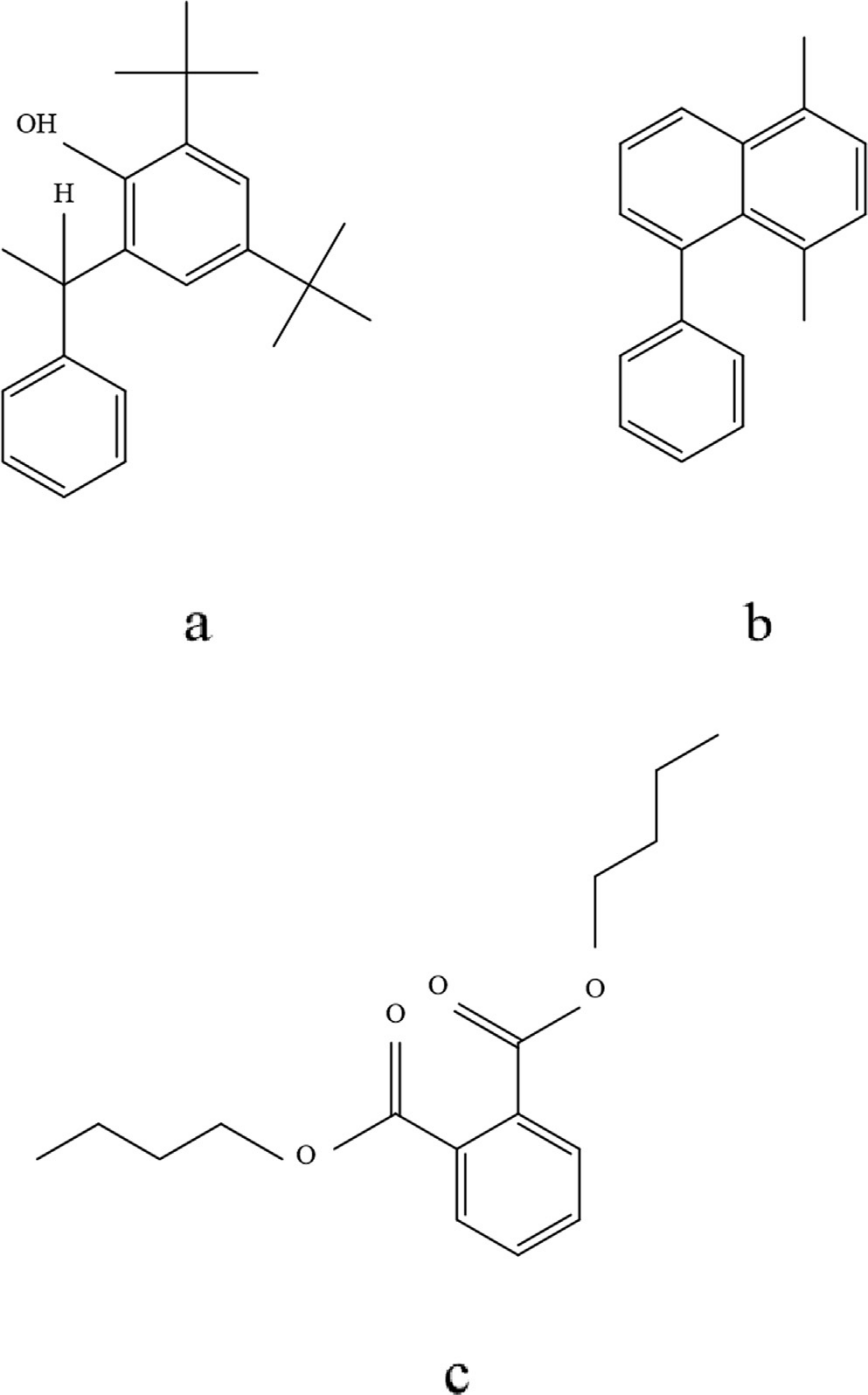
Major compounds detected in GC–MS analysis of *A. mollissima* leaves extract (**a**) 4,6-di-t-butyl-2-alpha-methyl benzyl phenol (**b**) 1,4-dimethyl-5-phenyl-naphthalene and (**c**) dibutyl phthalate*.*

The plant extract yielded ten identifiable compounds according to GC–MS analysis, shown in the chromatogram **(**Fig. 1**)** which were presented by their retention time (RT), molecular formula, molecular weight, and peak area (%). **(**Table 1**)** Among these, three major compounds with percent peak area were found to be 4,6-di-t-butyl-2-alpha-methyl benzyl phenol (10.58%), dibutyl phthalate (5.48%), 1,4-dimethyl-5-phenyl-naphthalene (3.89%). **(**Fig. 2**).**

**Table 1:** GC–MS data of compounds in *A. mollissima* leaves extract.

Sl. no.	RT	Phytochemicals	Molecular formula	Mol. wt. (gm/mol)	% Peak area
1	19.81	Pentanoic acid, 5-hydroxy-, 2,4-di-t-butyl phenyl esters	C_19_H_30_O_3_	306	1.11
2	21.041	Pentyl tetratriacontyl ether	C_39_H_80_O	564	0.94
3	27.323	Heptacosanoic acid, 25-methyl-, methyl ester	C_29_H_58_O_2_	438	1.15
4	29.823	Benzenepropanoic acid, 3,5-bis(1,1-dimethyl ethyl)-4-hydroxy-, methyl ester	C_18_H_28_O	292	1.53
5	30.802	Dibutyl phthalate	C_16_H_22_O_4_	278	5.48
6	31.694	10,18-Bisnorabieta-8,11,13-triene	C_18_H_26_	242	1.16
7	32.358	4,6-Di-t-butyl-2-alpha-methyl benzyl phenol	C_23_H_32_O	324	10.58
8	33.323	3,4-dihydro-2-(phenyl methylene)- 1(2H)-Napthalenone	C_17_H_14_O	234	0.78
9	33.497	2-Anilino-4-methyl quinoline	C_16_H_14_N_2_	234	1.43
10	34.409	1,4-Dimethyl-5-phenyl-naphthalene	C_18_H_16_	232	3.89

Upon quantitative analysis of the secondary metabolites, the extract exhibited the presence of a considerable quantity of phenolics, flavonoids and tannins. Total phenolic, flavonoid and tannin contents in the extract were obtained to be 142 mg GAE/g, 534 mg QAE/g and 110 mg GAE/g, respectively (Table 2).

**Table 2:** Approximate SC_50_ values of antioxidative assays and total secondary metabolites content of *A. mollissima* leaves extract.

Sample/ standard	DRSA (SC_50_ µg/ml)	HPSA (SC_50_ µg/ml)	TPC (mg GAE/g)	TFC (mg QE/g)	TTC (mg GAE/g)	TAC (mg AAE/g)
*A. mollissima* extract	60 ± 0.32	87 ± 0.25	142 ± 0.2	534 ± 0.016	110 ± 0.46	103 ± 1.2
Ascorbic acid	13 ± 0.21	40 ± 0.62				

*Values expressed are mean ± SD.

### 3.2 Antioxidative properties

In the TLC-based qualitative antioxidant assay, the presence of various UV and fluorescence positive components in *A. mollissima* extract was observed under UV light at 254 and 360 nm wavelength. Upon spraying DPPH solution, the visibility of yellowish spots on a reddish-purple background of the TLC plate suggested the presence of antioxidative components.

In the quantitative antioxidant assay, the extract revealed DPPH free radical scavenging activity with an SC_50_ value of 60 µg/ml, whereas the SC_50_ of the standard ascorbic acid was 13 µg/ml.

In the hydrogen peroxide scavenging assay, the extract displayed an SC_50_ value of 87 µg/ml compared to that of ascorbic acid (40 µg/ml).

While investigating the total antioxidant capacity of *A. mollissima* extract, it was reported to be 103 mg AAE/gm of dry extract.

### 3.3 Acute toxicity assay

**Fig. 3:**
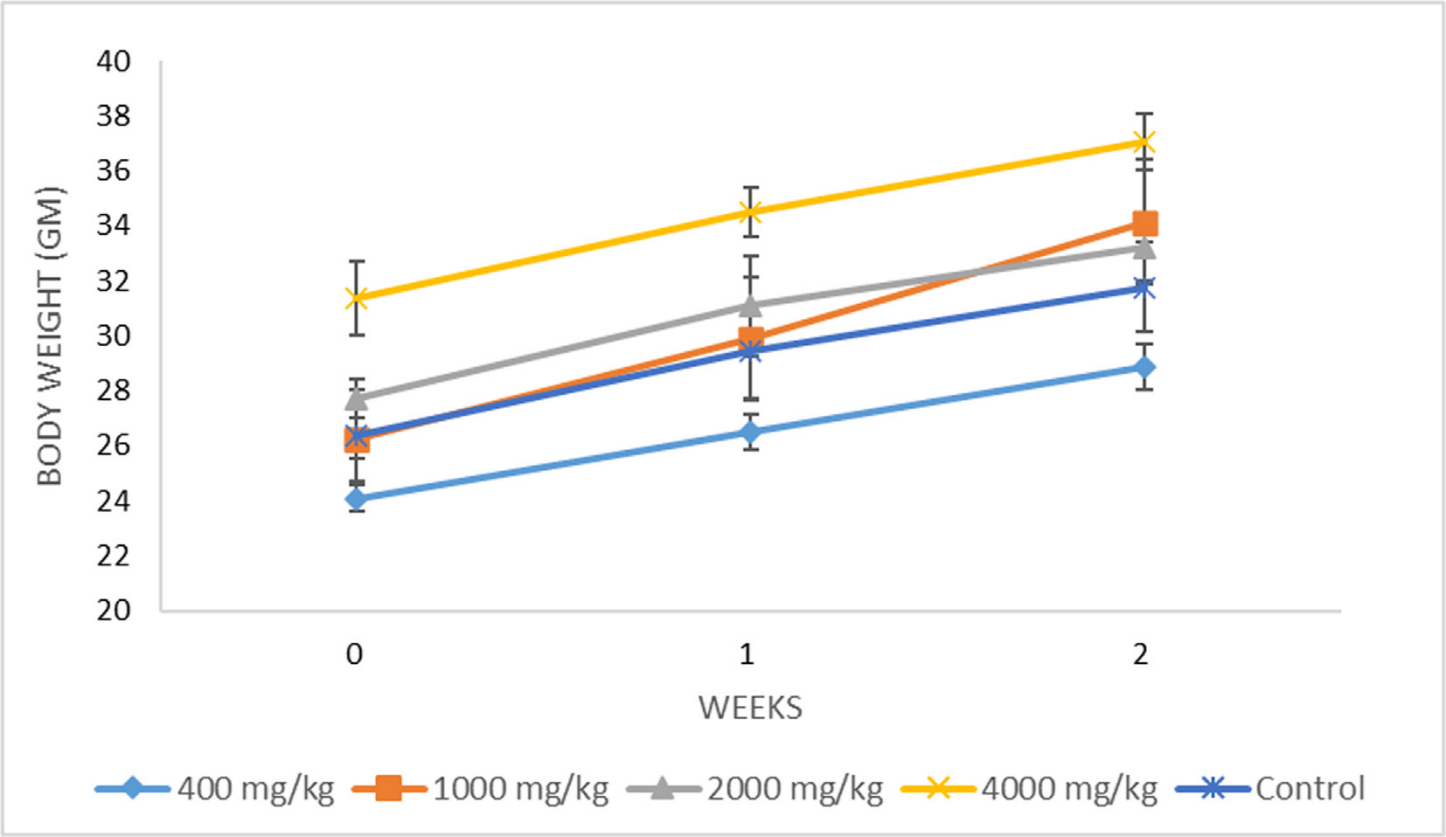
Effect of *A. mollissima* leaves extract on changes in body weight of mice.

No major changes in behavior, mortality, or body weight were seen in any of the treatment groups even at the highest dose of 4000 mg/kg bw. **(**Fig. 3**)** There were no symptoms of weight loss, salivation, anorexia, hypothermia, hyperthermia and abnormal body tone throughout the experimental period.

### 3.4 Analgesic activity study

In the measurement of analgesic activity, *A. mollissima* extract inhibited the writhing reflex significantly (p < 0.05) by 36% and 54% at doses of 250 and 500 mg/kg bw, respectively, and the diclofenac sodium was found to inhibit writhing response by 69% at a dose of 25 mg/kg bw. **(**Table 3**)**.

**Table 3:** Effect of *A. mollissima* leaves extract on acetic acid-induced writhing of mice (n = 5).

Animal group	Dose (mg/kg bw)	Mean writhing ± SD	% Inhibition of writhing	*t*-test (value of p)
Negative control	–	26.4 ± 2.79	**–**	**–**
Diclofenac Na	25	8.2 ± 1.64^*▲Δ^	68.94	12.56 (p < 0.0001)
*A. mollissima* extract	250	16.8 ± 4.49^*θ Δ^	36.36	4.07 (p = 0.0036)
*A. mollissima* extract	500	12.2 ± 2.16^*θ▲^	53.78	8.98 (p < 0.0001)

* p < 0.05 vs. Control (Dunnett’s *t* test); θ p < 0.05 vs. diclofenac Na 25 mg/kg bw; ▲ p < 0.05 vs *A. mollissima* extract 250 mg/kg bw; Δ p < 0.05 vs. *A. mollissima* extract 500 mg/kg bw (pair-wise comparison by Post Hoc Tukey test).

### 3.5 Antimicrobial activity assay

To test the antimicrobial potentiality of the extract, MICs against gram-negative and gram-positive bacterial strains were measured. The respective MICs of the extract against *E. coli, P. aeruginosa* and *S. aureus* were found to be 8, 16 and 8 µg/ml, while ciprofloxacin as the standard had MICs of 2, 1 and 4 µg/ml, respectively. **(**Table 4**)**.

**Table 4:** MIC (µg/ml) of *A. mollissima* leaves extract and standard against bacterial strains.

Bacterial class	Bacterial strain	*A*. *mollissima* extract	Ciprofloxacin
Gram-negative	*E. coli* *P. aeruginosa*	8 16	2 1
Gram-positive	*S. aureus*	8	4

### 3.6 Evaluation of antihyperglycemic activity

**Fig. 4:**
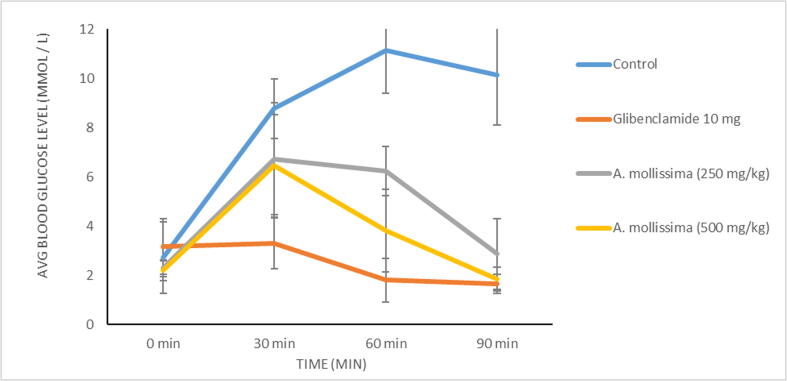
Effect of *A. mollissima* leaves extract and standard on the blood levels of glucose in mice group in OGTT.

At dosages of 250 mg/kg and 500 mg/kg bw, *A. mollissima* extract showed a drop in blood levels of glucose at 60 and 90 min in the OGTT to assess its antihyperglycemic action. (Fig. 4) But, there was no observed activity of the extract in the alpha-glucosidase enzyme inhibitory activity assay.

### 3.7 *In silico* analysis

**Fig. 5:**
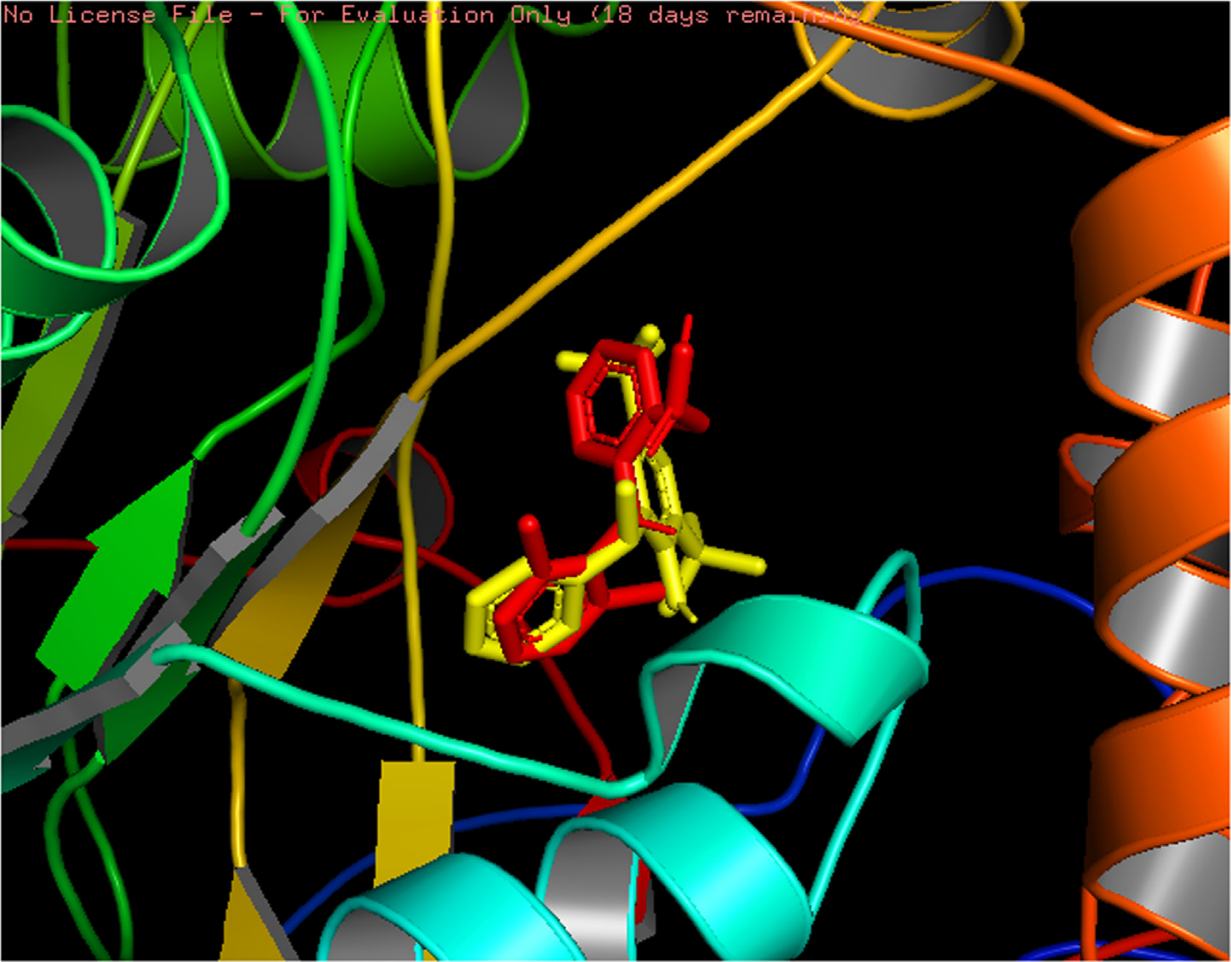
Binding region of compounds in the active site of 4NOS (diclofenac – red and 4,6-di-t-butyl-2-alpha-methyl benzyl phenol – yellow).

**Fig. 6:**
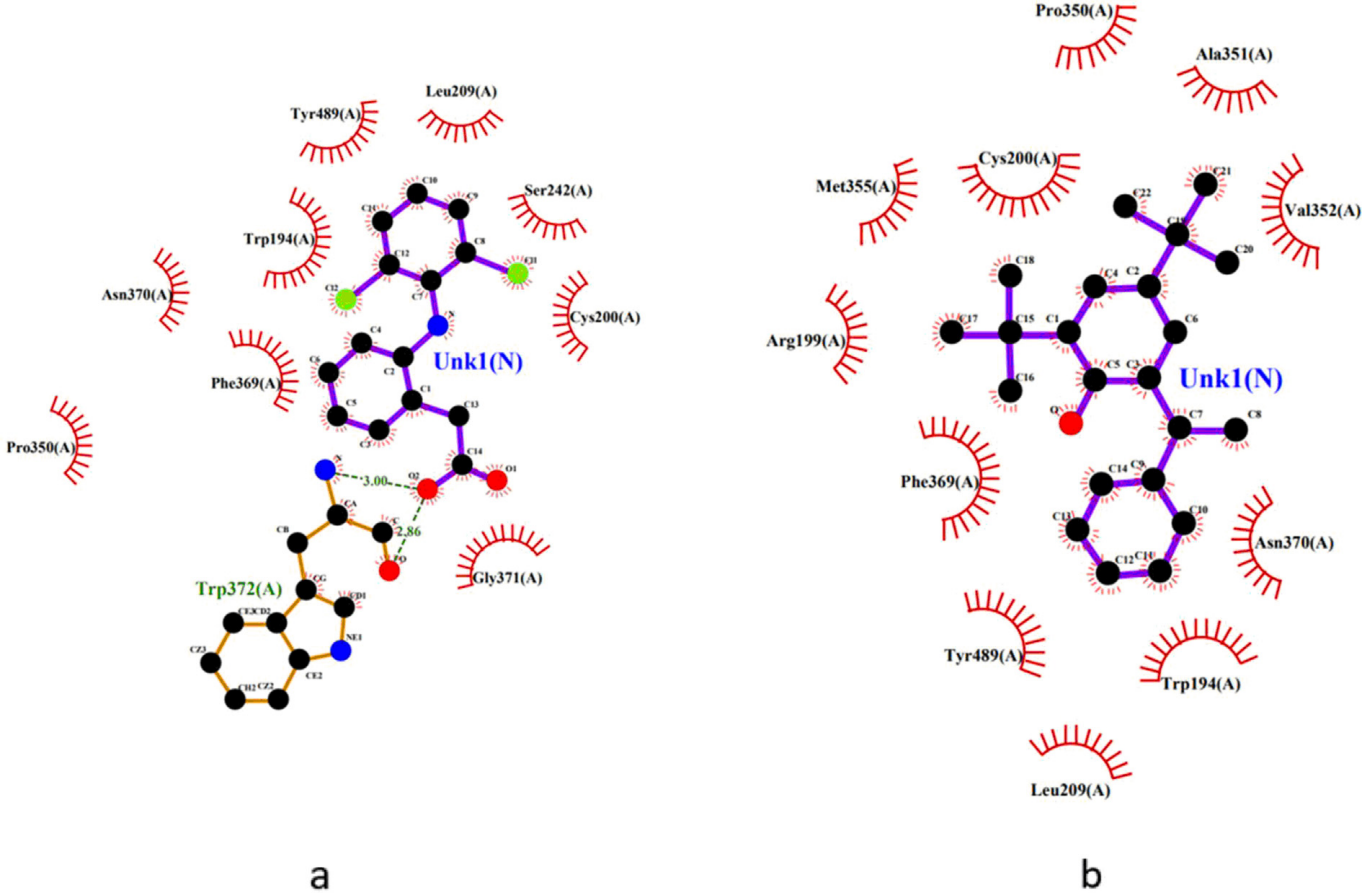
2D predicted binding mode of (**a**) diclofenac and (**b**) 4,6-di-t-butyl-2-alpha-methyl benzyl phenol with 4NOS receptor.

As potent *in vivo* analgesic and antihyperglycemic activity was observed, computer-aided studies were performed to further investigate these activities of the extract. An *in silico* analysis of the analgesic activity of the extract, 4,6-di-t-butyl-2-alpha-methyl benzyl phenol (Fig. 2a) exhibited a binding affinity of −9.9 kcal/mol which was better than standard diclofenac (−8.5 kcal/mol) and they were almost superimposable in their binding pockets. (Fig. 5) The binding mode predictions indicated that both of these interacted with the amino acids Leu209(A), Pro350(A), Cys200(A), Phe369(A), Asn370(A), Tyr489(A) and Trp194(A). (Fig. 6) The binding affinities of the other two compounds namely, 1,4-dimethyl-5-phenyl-naphthalene and dibutyl phthalate were −8.8 kcal/mol and −8.2 kcal/mol, respectively.

**Fig. 7:**
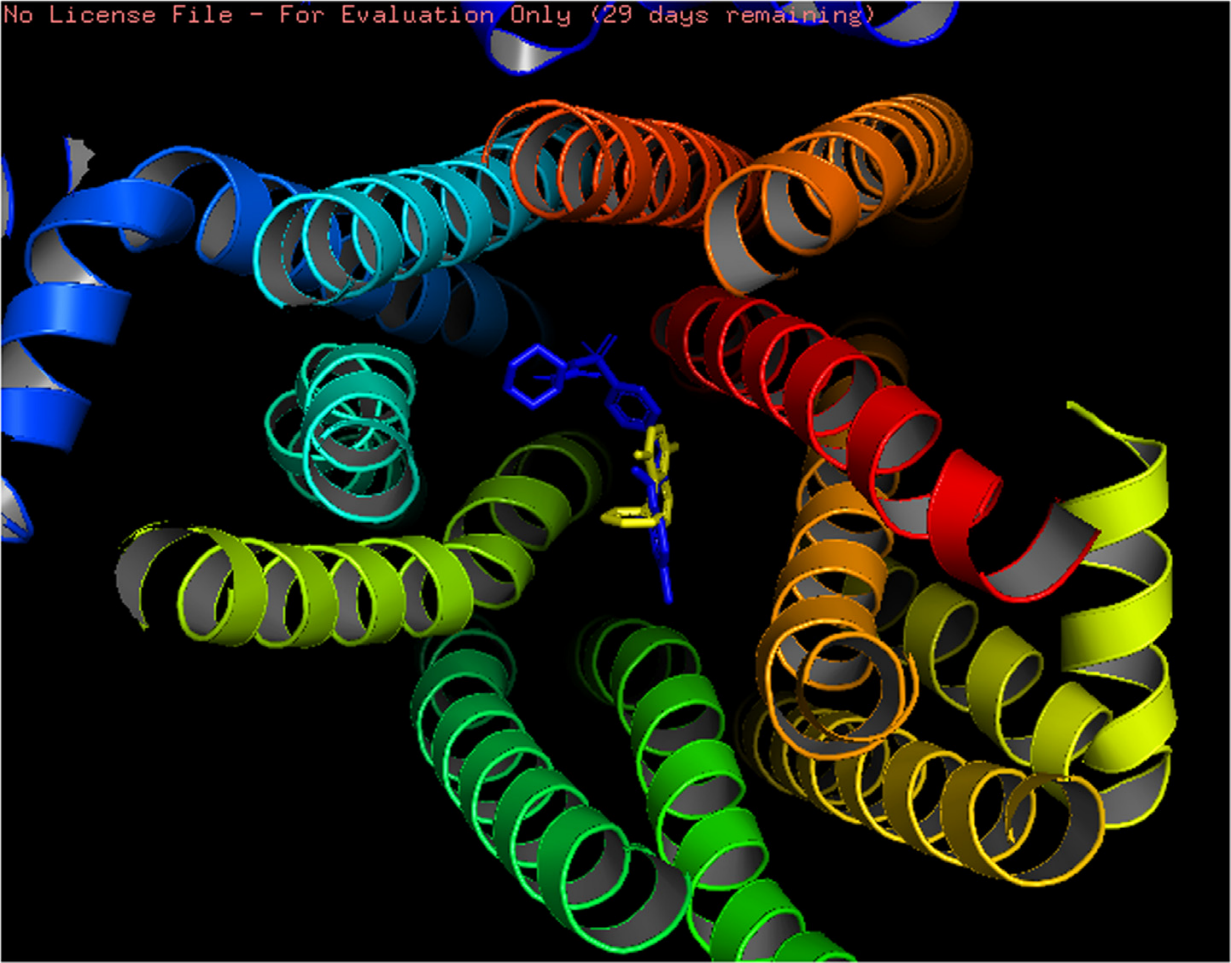
Binding region of compounds in the active site of 6JB3 (glibenclamide – blue and 1,4-dimethyl-5-phenyl-naphthalene – yellow).

**Fig. 8:**
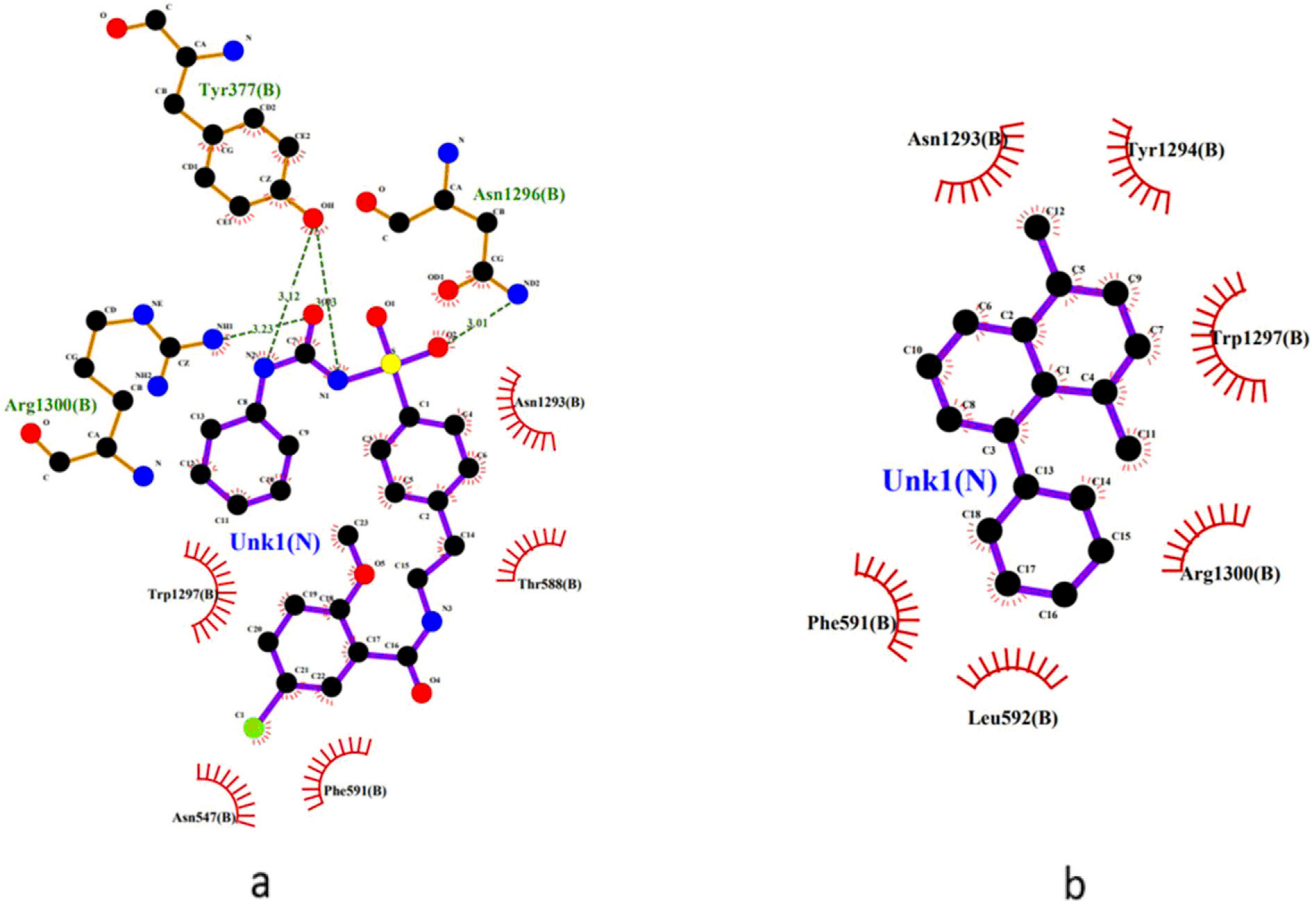
2D predicted binding mode of (**a**) glibenclamide and (**b**) 1,4-dimethyl-5-phenyl-naphthalene with 6JB3 receptor.

In molecular docking for validation of the antihyperglycemic activity of the extract, the compound 1,4-dimethyl-5-phenyl-naphthalene (Fig. 2b) revealed a binding affinity of −8 kcal/mol, which was close to that of the standard glibenclamide −8.8 kcal/mol. Here, the amino acids Asn1293(B), Trp1297(B), Arg1300(B) and Phe591(B) interacted with both glibenclamide and 1,4-dimethyl-5-phenyl-naphthalene. (Fig. 7, Fig. 8) The binding affinity of another major compound 4,6-di-t-butyl-2-alpha-methyl benzyl phenol was found to be −7.9 kcal/mol.

## 4 Discussion

Free radicals that are oxygen-centered and continuously produce different reactive oxygen species *in vivo* are implicated in cell death and tissue damage as well as several diseases including cancer, diabetes, heart diseases, aging, etc. (Shukla et al., 2009). Substances having the potential to protect the physiological system from oxidative stress induced by such free radicals are known as antioxidants (Ozsoy et al., 2008). Natural antioxidants present in plant extracts possess the ability to inhibit damaging effects induced by free radicals (i.e. reactive chemical species, noticeably hydroxyl radicals and superoxide ions (Majeed et al., 2021). Hence, it has now become important to identify natural antioxidants to combat a variety of physiological issues such as diabetes mellitus (Archana et al., 2021), atherosclerosis, chronic renal failure, cancer and immune dysfunction (Souri et al., 2010) as well as neurodegenerative diseases. As medicinal plants contain a mixture of chemical compounds, they are now considered an easily available and potent source of antioxidants (Saiah et al., 2016). In this study, phenols, flavonoids, glycosides, terpenoids, tannins and carbohydrates were found to be present in the phytochemical screening of *A. mollissima* extract, all of which play significant well-documented roles in biological activities, were indicative of the potential of the extract as a promising natural antioxidant.

The results from the DPPH free radical scavenging activity assay proved that *A. mollissima* extract was capable of donating an electron or hydrogen which could scavenge DPPH radical. Hydrogen peroxide is a non-radical oxidizing agent that passes through the cell membrane quickly and interacts with Fe^2+^ and Cu^2+^ ions for the formation of intracellular hydroxyl radicals (Mukhopadhyay et al., 2016). Phenolic compounds contain hydroxyl groups which enhance their free radical scavenging ability and induce them to act as antioxidants. Potential antioxidants can be screened rapidly as their total phenolic content can provide an estimate of antioxidative potential (Soobrattee et al., 2005). The phenolic compounds are quite popular regarding their wide range of biological effects as antimicrobial, antioxidant, anti-inflammatory and anticancer agents (Stefanucci et al., 2018). It has been found that *o*-benzyl-substituted phenols with two phenyl groups activating a methylene group have 1.5 or more times larger radical-trapping rate constants than the typical antioxidant butylated hydroxytoluene (BHT) (Matsuura and Ohkatsu, 2000). So, it can be inferred that the antioxidative potential displayed by the extract is most probably due to the presence of the *o*-benzyl-substituted phenol compound namely, 4,6-di-t-butyl-2-alpha-methyl benzyl phenol identified in the GC–MS analysis (Fig. 2a).

To predict the toxicity and determine a safe dose of plant products, toxicity studies are carried out in different experimental animals where usually, high dosages of the plant sample are administered in a single dose. As there is no previous study of *A. mollissima* in animal models, the acute toxicity test was performed, as per OECD guidelines, to assess its toxicity and determine the dose range that could be employed in future studies. In this study, the extract appears to be non-toxic at 4000 mg/kg bw, and the LD_50_ is estimated to be higher than the maximum dose applied.

As traditional use of *A. mollissima* most importantly included relief from headache and pain from injury, *in vivo* peripheral analgesic activity test was performed in this study. In the analgesic activity test, the writhing method was used in which irritant principle acetic acid was injected into mice to induce pain of peripheral origin (Gawade, 2012). In order to identify potential peripherally acting antinociceptive agents, this nociceptive model is quite efficient for dosages in which the analgesic and anti-inflammatory effects of drugs would be ineffective in other pain models (Nock et al., 2022). When acetic acid is administered by intraperitoneal injection, peripheral nociception is induced upon direct activation of non-selective cationic channels or indirect release of different endogenous mediators namely, prostaglandins, cytokines, bradykinin, along with increased production of the enzymes lipoxygenase (LOX) and cyclooxygenase (COX) (Alabi et al., 2019), thereby causing the stimulation of nociceptive neurons sensitive to the non‐steroidal anti-inflammatory drugs (Prabhu et al., 2011). Therefore, this test can be utilized to look into novel mild analgesic non-steroidal anti-inflammatory drugs. In this test, the analgesic effect of *A. mollissima* extract was comparable with that of diclofenac sodium which strongly recommends that extract of *A. mollissima* leaves have peripheral nociceptive activity and its mechanism of action may be mediated by a peripheral inhibition of LOX and/or COX, reduction in synthesis of prostaglandin and interference with the transduction mechanism in primary afferent nociceptors. Natural phenolic acids and flavonoids like rutin, quercetin, luteolin, etc. have been reported to have a primary role in analgesic activity upon targeting prostaglandins (Quibria et al., 2014). The analgesic potentiality of the extract could be due to the presence of flavonoids and phenolics in the phytochemical tests.

Because the leaves of *A. mollissima* have antiseptic property, *in vitro* antimicrobial activity assay was performed and the extract was found to have variable MICs against gram-positive as well as gram-negative strains. The compound dibutyl phthalate (Fig. 2c) reported by GC–MS analysis could be responsible for the significant antibacterial effect of the extract as previous studies showed that this compound has high antibacterial activity, especially against infectious diseases caused by *P. aeruginosa* (Khatiwora et al., 2012).

High antioxidative properties of this plant were seen in the preliminary screening of the plant rationalizing the carrying out of an antihyperglycemic test, as increased oxidative stress and low levels of antioxidants have been linked to diabetes (Golder et al., 2020). OGTT is carried out to measure the body's ability to utilize glucose and diagnose diabetes mellitus (Hoque et al., 2011). As the *A. mollissima* extract reduced the blood levels of glucose at 60 min and 90 min, the alpha-glucosidase enzyme inhibitory activity assay was performed to reveal the mechanism of this antihyperglycemic action. But the negative finding suggested some other mode of its antihyperglycemic activity rather than inhibiting the carbohydrate digesting alpha-glucosidase enzyme.

Computer-aided studies have unleashed a novel and innovative pathway toward drug discovery and development. By the virtual docking of a protein with the ligand to be investigated, we can obtain a better idea about the different biological activities of nature-derived molecules through their binding affinities to receptors and mechanisms of binding. To validate the results from the analgesic and antihyperglycemic activity tests performed in murine models, molecular docking was conducted utilizing the major compounds identified from extract using GC–MS. One of the major compounds 4,6-di-t-butyl-2-alpha-methyl benzyl phenol **(**Fig. 2a**)** showed a high binding affinity of −9.9 kcal/mol and previous studies have stated that phenolic derivatives have strong analgesic potential (Lee et al., 2006), so it corroborates the better binding affinity of 4,6-di-t-butyl-2-alpha-methyl benzyl phenol to the nitric oxide synthase (NOS) receptor responsible for inducing pain. In the molecular docking of the major compounds of the extract with the sulfonylurea receptor, 1,4-dimethyl-5-phenyl-naphthalene **(**Fig. 2b**)** and 4,6-di-t-butyl-2-alpha-methyl benzyl phenol had binding affinities ranging from −7.9 to −8 kcal/mol, both of which were very close to that of standard glibenclamide. Therefore, this antihyperglycemic activity of the extract might be attributed to the suppression of the sulfonylurea receptor by the above-mentioned compounds.

## 5 Conclusion

Upon GC–MS analysis of the ethanolic leaves of *A. mollissima*, 4,6-di-t-butyl-2-alpha-methyl benzyl phenol, dibutyl phthalate and 1,4-dimethyl-5-phenyl-naphthalene were obtained as three major compounds. The findings of this study evidenced that this plant has significant free radical scavenging activity and produces high levels of secondary metabolites that are responsible for many beneficial effects in the human body. In light of the acute toxicity study, no adverse effects of the extract were revealed in mice up to 4000 mg/kg daily. Analyzing the studied pharmacological activities, the extract was found to possess prominent analgesic, antihyperglycemic and antimicrobial effects thereby justifying the traditional usage of this plant. Further research into the pathways of these biological responses is essential, which can be performed by bioactivity-guided isolation of compounds.

## Declaration of Competing Interest

The authors declare that they have no known competing financial interests or personal relationships that could have appeared to influence the work reported in this paper.
